# Supplementary material for: Pyrogallol-Phloroglucinol-6,6-Bieckol from Ecklonia cava Attenuates Tubular Epithelial Cell (TCMK-1) Death in Hypoxia/Reoxygenation Injury
Source: Mar Drugs. 2019 Oct 24;17(11):602. doi: 10.3390/md17110602 (PMC6891818; doi:10.3390/md17110602)
Supplement: Supplementary file 1 [file marinedrugs-17-00602-s001.pdf]

Supplementary material

Table S1. List of antibodies for immunoblotting (Western blot)

Antibody name	Company	Cat. No.	Dilution rate
β-actin	Cell signaling	4967s	1:1000
NF-κB	Abcam	ab16502	1:500
TNF-α	Abcam	ab9739	1:500
IL-6	Abcam	ab6672	1:500
ERK1/2	Cell signaling	9102s	1:1000
Phospho-ERK 1/2 (pERK1/2)	Cell signaling	9101s	1:1000
SAPK/JNK	Cell signaling	9252S	1:1000
Phospho-SAPK/JNK (pSAPK/JNK)	Cell signaling	4668S	1:1000
P38	Cell signaling	8690S	1:1000
Phospho-p38 (pp38)	Cell signaling	4511S	1:1000

Figure S1. Experimental scheme images for of pre-hypoxia and post-hypoxia model *in vitro*

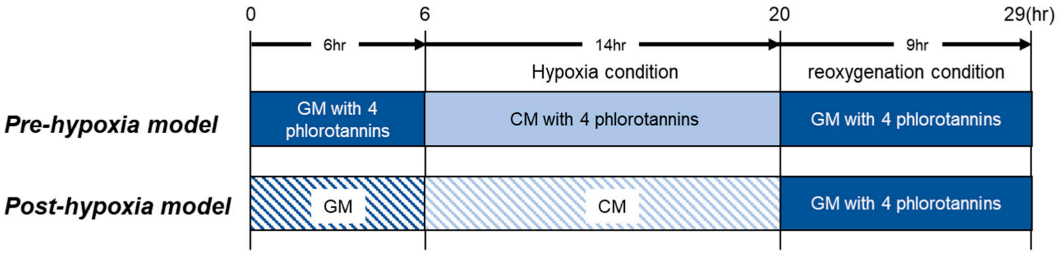

Experimental scheme showed in vitro methods of pre-hypoxia or post-hypoxia model. To make pre-ischemia model, four prepared phlorotannins (each concentration 2.5 μg/ml) were added to TCMK-1 cell growth medium (GM; 10% fetal bovine serum (FBS) and 1% penicillin/streptomycin in DMEM basal medium) for 6 h before ischemia. After phlorotannin treatment, the medium was completely removed, and the cells were washed thoroughly with PBS two time. Hypoxia conditioned medium (CM) contained 0.5% FBS and 1% penicillin/streptomycin in DMEM basal medium. The TCMK-1 cells were cultured with hypoxia condition (CM with the 4 phlorotannins in a mixture of 1% O2, 5% CO2, 94% N2) for 14 h. After hypoxia condition, the CM with the 4 phlorotannins was removed, and the cells were washed with PBS again. The cells were cultured with reoxygenation condition (GM with 4 phlorotannins in a mixture of 20% O2, 5% CO2, 75% N2) for 9 h, and then used for experiments. To make post-hypoxia model, TCMK-1 cells were cultured in GM for 6 h before hypoxia condition, GM was completely removed, and the cells were washed with PBS two times. Hypoxia condition was performed following above method. After finishing the hypoxia condition, the CM was removed, and the cells were washed with PBS again. The cells were cultured with reoxygenation condition for 9 h, and then used for experiments.
